# Diagnosis of Prosthetic Endocarditis Caused by *Coxiella burnetii* Using PET Scan and PCR: A Case Report of Chronic Q Fever

**DOI:** 10.1002/ccr3.70289

**Published:** 2025-03-05

**Authors:** Sara Ghaderkhani, Maryam Moradi, Mahsa Azadbakhsh kanaf gorabi, Fereshteh Ghiasvand, Farnoosh Larti, Saber Esmaeili, Ensiyeh Rahimi

**Affiliations:** ^1^ Department of Infectious Diseases and Topical Medicine, School of Medicine, Imam Khomeini Hospital Complex Tehran University of Medical Sciences Tehran Iran; ^2^ Eye Research Center, The Five Senses Health Institute, Rassoul Akram Hospital Iran University of Medical Sciences Tehran Iran; ^3^ Liver Transplantation Research Center, Department of Infectious Diseases, Imam Khomeini Hospital Complex Tehran University of Medical Sciences Tehran Iran; ^4^ Department of Cardiology, School of Medicine, Prehospital and Hospital Emergency Research Center, Imam Khomeini Hospital Complex Tehran University of Medical Sciences Tehran Iran; ^5^ Department of Epidemiology and Biostatistics, Research Centre for Emerging and Reemerging Infectious Diseases Pasteur Institute of Iran Tehran Iran; ^6^ Iranian Research Center for HIV/AIDS, School of Medicine, Imam Khomeini Hospital Complex Tehran University of Medical Sciences Tehran Iran

**Keywords:** blood culture‐negative endocarditis, chronic endocarditis, *Coxiella burnetii*, Q fever

## Abstract

Chronic blood culture‐negative endocarditis (BCNE) presents a significant challenge for early diagnosis and treatment, leading to increased morbidity and mortality. This report presents a 30‐year‐old man with a history of BCNE who presented with an intermittent fever lasting 3 months. His medical history was complex and characterized by tetralogy of Fallot (TOF), multiple cardiac surgeries, and previous positive pathological results for infection and endocarditis. A PET/CT scan revealed hypermetabolic lesions near the prosthetic valves and aortic grafts, prompting further investigation for potential causative organisms. Subsequent serological testing and PCR confirmed the presence of *Coxiella burnetii* , leading to a diagnosis of Q fever endocarditis. Treatment with doxycycline and hydroxychloroquine initiated significant improvement. Follow‐up after 3 months showed that the patient remained stable with significant improvements in serological tests and imaging. This case underscores the necessity of considering atypical pathogens like *C. burnetii* in patients with BCNE and chronic endocarditis, particularly those with complicated cardiac histories.

**Summary**

- In patients with blood culture‐negative endocarditis and complex cardiac conditions, consider atypical pathogens like *Coxiella burnetii* , especially in endemic areas.
- Advanced imaging (e.g., PET/CT) and thorough serological testing and PCR are crucial for diagnosis and management, particularly in persistent fever cases, to reduce morbidity and mortality.

## 1 Introduction

Infective endocarditis (IE) poses a significant clinical challenge, with approximately 30 new cases reported per million people worldwide per year [1]. Early diagnosis and appropriate treatment are crucial due to its potentially fatal outcomes [1]. IE can arise from valvular conditions, prior heart surgeries, and the presence of implanted devices, leading to both cardiac and systemic complications. Early detection utilizing laboratory tests, transesophageal echocardiography (TEE), imaging, and cultures is essential for adequate patient management [2].

A notable diagnostic challenge is blood culture‐negative endocarditis (BCNE), which accounts for approximately 20% of IE cases despite advances in diagnostic techniques [3]. The organisms typically implicated include Bartonella, Coxiella, and Chlamydia species [4], with Bartonella being the leading cause in recent years [5].

Q fever, caused by the zoonotic pathogen *Coxiella burnetii* , is endemic in parts of Iran due to extensive contact with domestic animals, presenting a significant consideration for BCNE in these regions [6]. This report details a patient with Q fever endocarditis and a history of chronic endocarditis and multiple valve replacements.

## 2 Case History

A 30‐year‐old man was admitted with intermittent fever persisting for 3 months. On admission, his temperature was 38°C, with normal vital signs. Notable physical findings included mild cyanosis of the lips, hyperpigmentation (lipodermatosclerosis) in both legs, splenomegaly, and a mid‐systolic murmur.

He resided in a rural area and had possible indirect contact with goats. He reported no recent direct animal contact or suspicious exposures, aside from occasionally consuming local cheese, noting that he was the only febrile individual in his household.

The patient had a history of BCNE episodes at ages 25 and 27, along with tetralogy of Fallot. His surgical history included a Blalock‐Taussig shunt in 1994, total correction of TOF in 2001, and bioprosthetic pulmonary valve replacement in 2010.

In 2015, due to infective endocarditis of the aortic valve, the patient underwent the Benthal operation for the native aortic valve along with mechanical pulmonary valve replacement.

In the previous episode of endocarditis in 2019, transesophageal echocardiography revealed a suspicious semi‐mobile mass in the distal ascending aorta tube graft. Despite empirical antibiotic treatment for endocarditis, the patient's symptoms persisted, and he remained febrile. Due to the high surgical risk and inconclusive echocardiographic findings, the surgical team initially refused to operate. However, a PET/CT scan, conducted after significant delays due to insurance issues, indicated increased metabolic activity in the aortic root and tubular graft, suggestive of active inflammation or infection. The patient subsequently underwent surgery, revealing extensive involvement of the tubular graft but no pseudoaneurysm or prosthetic valve infection. He received a Bentall homograft procedure and continued antibiotic treatment for 6 weeks. Postoperatively, his platelet count normalized, corticosteroids were discontinued, and at one‐year follow‐up, he was asymptomatic with normal inflammatory markers and echocardiographic findings [7]. After 9 months, the patient's intermittent fevers started. In each episode of endocarditis, blood cultures were negative, and she received antibiotics for culture‐negative endocarditis.

In the re‐examination and PCR performed on the pathology sample from 2015, *Coxiella burnetii* was detected, indicating chronic endocarditis due to the patient's heart complications, including an artificial valve (Table 1).

**TABLE 1:** Clinical characteristic, progression, and treatment.

**Patient overview** – Age: 26 years – Traumatic knee injury (June 2023) – Pain and swealing in the left knee			**Past medical history** – Common variable immunodeficiency (CVID) – History of autoimmune hemolytic anemia and rheumatoid arthritis – Intravenous immunoglobulin (IVIG) therapy			
**Synovial fluid analysis**	Color	Appearance	Glucose	WBC	Poly	Culture
6 August 2023	Yellow	Turbid	61	10,000	20%	Neg.
Not initially considered septic arthritis						
6 December 2023	Milky	Turbid	30	46,000	90%	Neg.
**Diagnosis of septic arthritis (July 2023)** Treatment initiated: (Incomplete treatment) – Vancomycin (10 days) – Cefepime (10 days) – Arthrotomy and repeated arthrocentesis – Negative cultures			**Recurrent symptoms (July to December 2023)** – Multiple joint lavages – PCR for TB and brucellosis negative – Treatment with vancomycin and cefepime			
**MRI findings in November 2023:** – Moderate joint effusion – Synovial thickening with enhancement – Edema and inflammation has spread to the soft tissues						
**Diagnosis** – PCR: *Mycoplasma hominis* (in 25 December 2023)			**Treatment initiated** – Medications: – Doxycycline (100 mg every 12 h) – Moxifloxacin (400 mg once daily)			
**Response to treatment:** – Fever subsided by 5th day – CRP levels decreased from 90 to 20 after 1 week **Follow‐up:** – Discharge: Patient discharged in stable condition – Serological monitoring: Follow‐up every 3 months – Continued antibiotic regimen: For 30 months **Three month follow‐up findings:** – Normalization of inflammatory markers: ESR and CRP – Improved serological tests – Improved PET/CT results **Tree years follow‐up findings:** – Normalization of inflammatory markers: ESR and CRP – Improved serological tests						

Given his BCNE history and ongoing fever, further investigations were warranted. Initial lab findings indicated a normal leukocyte count (WBC 5700 × 10^9/L^), anemia (Hb 12.1 g/dL), thrombocytopenia (124,000 platelets), elevated creatinine (2 mg/dL), and significantly elevated inflammatory markers (ESR 120 mm/h, CRP 31 mg/L). Urinalysis, viral markers, Coombs test, 2‐mercaptoethanol (2ME) Brucella agglutination test, and six blood cultures yielded negative results.

Electrocardiogram (ECG) findings included right axis deviation and various degrees of AV block. Chest CT showed no abnormalities, and TEE failed to reveal any indications of IE; however, the ejection fraction was noted at 40%. Aortic and pulmonic prosthetic valves had normal pressure gradients with no evidence of paravalvular regurgitation or obvious vegetation.

**FIGURE 1:**
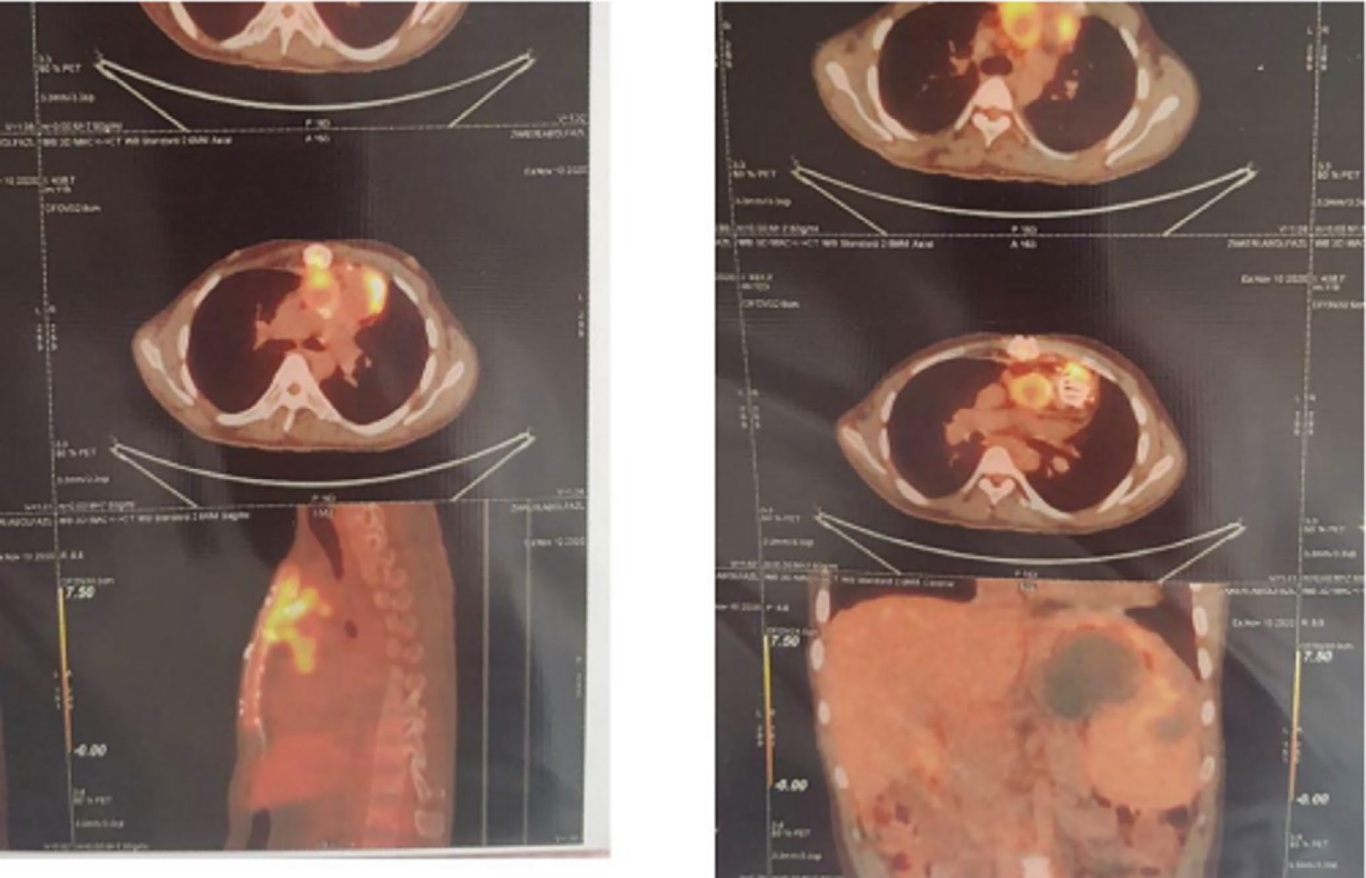
PET/CT scan: Hypermetabolic lesions around the aortic and pulmonary prosthetic valves, as well as involvement of the ascending aorta and proximal pulmonary artery.

Based on the previous BCNE history and PET/CT involvement, a PET/CT scan was conducted, revealing hypermetabolic lesions around the aortic and pulmonary prosthetic valves, as well as involvement of the ascending aorta and proximal pulmonary artery. Moreover, the sternum showed increased metabolic activity, suggestive of possible osteomyelitis, with right sub‐pectoral adenopathy. Modified Duke's Criteria supported a clinical suspicion of endocarditis (Figure 1).

Surgery is recommended in patients with congestive heart failure, prosthetic valve endocarditis, and uncontrolled infection [8].

## 3 Differential Diagnosis, Investigations, and Treatment

Following the PET/CT scan, further examinations for pathogens causing BCNE were pursued, with a focus on brucellosis and Q fever. Samples were sent to the National Laboratory for Plague, Tularemia, and Q fever. Real‐time PCR and IFA tests confirmed positive results for *Coxiella burnetii* (IgG phase I: 1:16384, IgG phase II: 1:16384), validating the diagnosis of Q fever endocarditis. Previous samples from the recent valve replacement also tested positive for *C. burnetii* .

The patient was commenced on doxycycline (100 mg every 12 h) and hydroxychloroquine (200 mg every 8 h). The patient exhibited a positive response to treatment, with fever subsiding by the 5th day. C‐reactive protein (CRP) levels significantly decreased from 90 to 20 after 1 week, indicating a reduction in inflammation (Table 1). The patient was discharged in stable condition, suggesting that their clinical status had improved sufficiently to allow for outpatient management.

## 4 Conclusion and Results

A serological monitoring plan was established, with follow‐up appointments scheduled every 3 months to assess ongoing health and treatment response. The patient was prescribed a continued antibiotic regimen for a total duration of 30 months to ensure complete management of the underlying condition.

During the three‐month follow‐up, there were notable improvements. Both erythrocyte sedimentation rate (ESR) and CRP levels returned to normal ranges. Imaging studies demonstrated positive changes. Sternum uptake on PET/CT improved significantly.

At the three‐year follow‐up, the patient continued to show favorable outcomes. ESR and CRP levels remained within normal limits, indicating sustained control of inflammation. Ongoing serological assessments showed continued improvement, reinforcing the effectiveness of the treatment regimen. The patient currently remains asymptomatic.

This case underscores the necessity of considering atypical pathogens like *C. burnetii* in patients with BCNE and chronic endocarditis, particularly those with complicated cardiac histories.

## 5 Discussion

In our study, similar to findings in other studies, Q fever often presents with chronic complications such as endocarditis, which is associated with significant morbidity and mortality. While patients may exhibit various non‐specific symptoms, chronic Q fever is particularly challenging to diagnose and manage due to antibiotic resistance and the propensity for relapse. Surgery on abnormal valves heightens the risk for chronic Q fever, as seen in our patient [8]. Surgery is recommended in patients with congestive heart failure, prosthetic valve endocarditis, and uncontrolled infection [9]. Based on this, the surgery was not included in our patient's condition.

Given the endemic presence of *C. burnetii* in many regions, it is vital to consider it a potential agent for BCNE. Even with medical advancements, IE remains a critical issue in both diagnosis and treatment, contributing to a mortality rate approaching 30% annually. TEE provides an effective, non‐invasive method for detecting vegetations, yet can miss cases, as demonstrated in our patient with normal echocardiographic results despite significant clinical suspicion [10].

To discern extra‐cardiac manifestations of IE, PET/CT scans serve as a valuable diagnostic tool. In this case, conventional imaging studies yielded no significant findings, but PET/CT provided crucial insights indicative of infective endocarditis [11].

Sternal osteomyelitis is an infection affecting the sternum, which can arise as a complication following several medical procedures, especially cardiac surgeries or invasive chest procedures like sternotomy. In cases related to endocarditis, this condition is frequently linked to the dissemination of infection from the heart valves to adjacent areas, including the sternum [12]. Sternal wound infection and sternal osteomyelitis incidence vary between 0.5% and 8% of patients undergoing cardiac surgery [13]. FDG PET/CT is a diagnostic tool in several infectious diseases, such as prosthetic joint infections, vertebral osteomyelitis, vascular prosthesis infection, and cardiac implantable electronic devices (CIED) infection [14, 15]. In cases related to endocarditis, this condition is frequently linked to the dissemination of infection from the heart valves to adjacent areas, including the sternum.

The integration of PET/CT scans with echocardiography has enhanced the sensitivity for diagnosing infective endocarditis (IE) and infections related to aortic grafts, and it has the potential to reclassify patients with ambiguous diagnoses [16]. However, it is important to note that the specificity of PET scans is not particularly high. Consequently, non‐infectious inflammatory conditions, such as post‐surgical inflammation occurring within a few months after cardiac surgery, may lead to false‐positive results when this imaging modality is utilized [17].

According to a systematic review and meta‐analysis study in Iran, the estimated seroprevalence of Q fever among humans was 19.8, respectively [18]. Considering the existence of Iran, it should always be considered.

This case underscores the necessity of considering atypical pathogens like *C. burnetii* in patients with BCNE, particularly those with complicated cardiac histories. The lack of identification of the pathogen through standard diagnostic methods often results in inappropriate antibiotic treatment, increasing morbidity and mortality risks.

Q fever‐induced endocarditis frequently lacks specific clinical manifestations and echocardiographic evidence, delaying timely diagnosis and appropriate treatment. This oversight can lead to numerous complications, contributing to increased morbidity and mortality rates. Given the endemic nature of Q fever in various geographical regions, clinicians should maintain a high index of suspicion for this diagnosis in patients with histories of animal contact, especially those with complex cardiac histories. Access to laboratory tests and the use of rapid diagnostic methods will confer significant advantages in effectively managing such cases. Multimodal imaging, particularly PET/CT, is an essential component of modern diagnostic strategies in suspected cases of endocarditis.

## Author Contributions

**Sara Ghaderkhani:** conceptualization, project administration, writing – review and editing. **Maryam Moradi:** formal analysis, methodology, validation. **Mahsa Azadbakhsh kanaf gorabi:** investigation, methodology. **Fereshteh Ghiasvand:** conceptualization, supervision, validation. **Farnoosh Larti:** conceptualization, visualization. **Saber Esmaeili:** formal analysis, validation. **Ensiyeh Rahimi:** data curation, supervision.

## Ethics Statement

This study has been approved by the ethics committee of Tehran University of Medical Sciences and adheres to the Declaration of Helsinki, with informed consent obtained from the patient for publication.

## Consent

The consent form is signed by the patient. A Written informed consent was obtained from the patient to publish this report in accordance with the journal's patient consent policy.

## Conflicts of Interest

The authors declare no conflicts of interest.

## Data Availability

Data sharing not applicable—no new data generated, or the article describes entirely theoretical research.
